# Efficient generation of human dorsal spinal GABAergic progenitors for the treatment of spinal cord injury

**DOI:** 10.1038/s12276-026-01665-8

**Published:** 2026-03-06

**Authors:** Xianglan Feng, Yutong Wan, Minxian Peng, Yan-Ting Cheung, Zhinan Lin, Kin-Wai Tam, Chaoyang Fan, Yongting Yang, Dengcheng Zhan, Huazhang Zhu, Ying Yu, Xifeng Wang, Qiang Liu, Xiaowei Zhu, Ying-Shing Chan, Martin Cheung, Chi-Wai Cheung, Jessica Aijia Liu

**Affiliations:** 1 Department of Anaesthesiology, School of Clinical Medicine, Li Ka Shing Faculty of Medicine, The University of Hong Kong, Hong Kong, China; 2 Department of Neuroscience, College of Biomedicine, City University of Hong Kong, Hong Kong, China; 3 Department of Physiology and Biophysics, Jacobs School of Medicine and Biomedical Sciences, State University of New York, Buffalo, NY USA; 4 Department of Anesthesiology, the First Affiliated Hospital, Jiangxi Medical College, Nanchang University, Nanchang, China; 5 School of Biomedical Sciences, Li Ka Shing Faculty of Medicine, The University of Hong Kong, Hong Kong, China

**Keywords:** Transdifferentiation, Spinal cord injury

## Abstract

Traumatic spinal cord injury (SCI) induces rapid necrotic cell death, leading to severe neuronal and glial loss. A critical consequence is the disruption of γ-aminobutyric acid (GABA)ergic inhibitory tone in the dorsal horn, which results in excessive glutamate release and neuronal hyperexcitability—a hallmark of central neuropathic pain and excitotoxicity that exacerbates secondary spinal damage. Notably, GABA itself exhibits neuroprotective properties, mitigating secondary injury and promoting neurite outgrowth during development or after central nervous system trauma. Whereas human neural stem cell-based therapies hold promise for compensating neuronal loss after SCI, their efficacy is limited by the hostile injury microenvironment and default differentiation pathways, which restrict the generation of mature, dorsal spinal GABAergic neurons. Here we identified key transcription factors that rapidly convert human pluripotent stem cells into dorsal spinal GABAergic progenitors with high efficiency. These induced GABAergic progenitors demonstrated remarkable resilience in the injured microenvironment, exhibiting intrinsic capacity to generate mature GABAergic neurons for functional integration. Importantly, they also exert noncell autonomous effects, reducing apoptosis, inhibiting glial scar formation and stimulating neurogenesis of endogenous cells following SCI. On integration into host neural circuitry and niche rewiring, induced GABAergic progenitor grafts significantly improved central neuropathic pain as early as 6 weeks after grafting and enhanced locomotor activities, demonstrating their great potential for future clinical applications in SCI treatment.

## Tailored GABAergic neuron therapy repairs spinal cord injury

Traumatic spinal cord injury often leads to movement problems and central neuropathic pain owing to the loss of inhibitory GABAergic neurons. Researchers aimed to address this by exploring new strategies to generate these specific types of neurons that could help alleviate these issues. Researchers used a method called transcription factor programming to effectively convert human pluripotent stem cells into GABAergic neurons with a dorsal spinal identity, meaning they match the type of neurons found in the spinal cord. When transplanted into rats with spinal cord injury, these newly generated neurons integrated well into the host tissue, reduced pain and improved movement. This suggests that these programmed neurons could be a promising cell therapy for treating spinal cord injury and related complications.

This summary was initially drafted using artificial intelligence, then revised and fact-checked by the author.

## Introduction

Traumatic spinal cord injury (SCI) is one of the leading causes of disability, resulting in permanent sensory and motor deficits, and is often accompanied by debilitating complications, such as central neuropathic pain (CNP)^1^. Affecting over 50% of patient with SCI, CNP manifests as allodynia (pain behavior evoked by non-noxious stimuli) and hyperalgesia (exaggerated pain behavior evoked by noxious stimuli), severely impacting the quality of life of patients. Current treatments remain ineffective, and sustained CNP can lead to irreversible nociceptive system changes^2^. Dysfunction or loss of γ-aminobutyric acid (GABA)ergic interneurons in the dorsal horn resulting from injury causes diminished GABA release, which disinhibits excitatory glutamatergic interneurons, leading to neuronal hyperexcitability, a key mechanism that induces CNP development^3–6^. In addition, excitotoxic levels of glutamate after SCI contribute to the widespread death of neurons and oligodendrocytes^7^, leading to progressive secondary injury. GABA also exerts neuroprotective properties, which not only mitigate excitotoxicity by inactivating descending dorsal interneurons caused by excessive glutamate but also promote neurite outgrowth during development or after central nervous system damage^8,9^. Therefore, restoring the functional GABAergic neuronal properties in the dorsal spinal cord presents a promising therapeutic strategy for SCI to address further damage and its associated complications, particularly CNP.

Given the limited regenerative capacity of the adult spinal cord, the transplantation of desired cell types derived from human pluripotent stem (PS) cells offers a promising strategy for spinal repair after injury. Transplantation of forebrain GABAergic progenitors was shown to enhance GABAergic signaling in the spinal cord, attenuating neuropathic pain in an SCI mouse model^10^. However, the presence of mixed cell types that do not match the recipient’s spinal tissue limits the clinical applicability and complicates the spinal microenvironment in the long term. Moreover, the injured spinal cord’s hostile microenvironment often diverts human neural stem cells (hNSCs) toward astrocytic or excitatory neuronal fates, yielding few functional GABAergic neurons while promoting glial scar formation^11–13^. Excitotoxic glutamate levels further exacerbate this astrocytic bias^7^. To effectively treat CNP and further damage arising from SCI, there is a need for methods that can generate a relatively high-purity population of GABAergic neurons with dorsal spinal identity, enhancing clinical translation.

Recent advances in cellular reprogramming have enabled the direct conversion of non-neural cells into specific neuronal subtypes, including forebrain GABAergic neurons^14–16^. However, generating dorsal spinal GABAergic neurons, which are under distinct developmental signaling control, remains underexplored. Here, we identified key transcription factor (TF) combinations to directly convert human PS cells into dorsal spinal GABAergic progenitors effectively. These TF-induced GABAergic progenitors (iGABAPs), characterized by their matched dorsal spinal identity, effectively counteract the detrimental effects of the injured microenvironment with remarkable resilience in generating mature GABAergic neurons. Surprisingly, TF-induced GABAergic cells also exert noncell autonomous effects in improving the injured niche, which dramatically reduces apoptosis and promotes survival and neurogenesis of endogenous cells following SCI. Critically, Integration of iGABAPs graft into host neural circuits significantly alleviates CNP within 6 weeks after grafting and enhances locomotor recovery, highlighting their therapeutic potential for SCI.

## Materials and methods

### Pluripotent stem cell culture

Human PS cells (IMR90, H9 and HES2) were provided by WiCell Research Institute, at passages 33–49. Cells were cultured on Matrigel gel (Corning)-coated plates in mTeSR (Stem Cell Technologies) or StemFlex (Thermo Fisher Scientific). Cells were passaged with ReLeSR (Stem Cell Technologies), washed and replated at a dilution of 1:5 or 1:10.

### Plasmids and virus particles

We used a doxycycline (dox)-inducible lentiviral vector (FUW-TetO-T2A-blastidicine) backbone for overexpressing TFs BRN2, LBX1 and PAX2, respectively. A lentiviral vector pLVX-EF1α-puro (Clontech)^17^ under the EF1a promoter with puromycin selection was used for overexpressing PTF1A and ASCL1, respectively. To generate lentiviral particles, 293 T cells were transfected with a lentiviral expression vector, packaging plasmid psPAX.2 and envelope plasmid pMD2.G using PolyJet (SignaGen). The cell culture medium containing the lentiviral particles was collected at 48 and 72 h after transfection, filtered through a 0.45-μm filter. The lentiviral particles were titrated using a Lenti-X qRT-PCR Titration Kit (Takara). Next, 3 × 10^5^ human PS cells were infected with quantified lentivirus particles expressing complementary DNA and/or short hairpin RNA and cultured in the presence of 8 μg/ml Polybrene (Sigma) for 24 h. After 48 h of transduction, infected cells were screened in the presence of 1 μg/ml puromycin (Life Technologies).

### Generation of TF-induced GABAergic neurons

To generate iGABAPs, human PS cells were dissociated with ReLeSR and plated on 12-well plates (about 3 × 10^4^ cells per well) coated with Matrigel. Cells were cultured in StemFlex containing Y-27632 (1 μM). Lentivirus produced as described above was added to fresh StemFlex medium, and cells were treated with blasticidine and/or puromycin for 5–7 days in N2B27 medium containing retinoic acid (RA; 0.5 μM), EGF (20 ng/ml), FGF (20 ng/ml) and dox (2 mg/ml). We started to observe colony formation after 7–14 days of treatment and further picked colonies at 21 days of treatment for subsequent neuronal differentiation, in vivo grafting and analysis.

### Generation of dorsal spinal cord neural progenitors

To induce dorsal spinal neuroepithelia formation, human PS cells were suspended on a low-attachment plate for 2 days in neural induction medium and then plated on Matrigel-coated plates as previously described^18^. Neural induction medium is composed of N2B27 medium with 250 nM LDN-193189 (Tocris Biosciences), 10 μM SB-431542, 2 μM DMH1, 3 μM CHIR-99021 (Tocris Biosciences), 0.5 μM cyclopamine and 250 nM RA (Tocris Biosciences). The medium was replaced every 2 days. On days 7–9, neuroepithelial cells formed neural tube-like rosettes, which were gently selected using a 200-μl pipette on day 10 and cultured with neural maintenance medium with cyclopamine (0.5 μM), and 250 nM RA for the first 7 days in Matrigel-coated wells or a low attachment plate. For neural maintenance medium, N2B27 medium was supplemented with 1 μM cyclic adenosine monophosphate (cAMP) (Sigma-Aldrich), 20 ng/ml EGF and 20 ng/m FGF (Peprotech). For neuronal differentiation, dorsal neural progenitors were cultured in neuronal differentiation medium for 7–35 days. Neuronal differentiation medium is composed of N2B27 medium supplemented with 20 ng/ml BDNF, 20 ng/ml GDNF and 20 ng/ml IGF. For neural medium used to assay the spontaneous differentiation capacity of TF reprogramming, N2B27 medium was supplemented with 1 μM cAMP (Sigma-Aldrich), 20 ng/ml EGF and 20 ng/ml FGF (Peprotech) without any other trophic factors.

### Immunocytochemistry

Cultures were fixed for 30 min in 4% paraformaldehyde in 0.1 M phosphate buffer at 4 °C. After washing three times with PBS, fixed samples were permeabilized with 0.1% Triton X-100 with 1% BSA in PBS for 1 h at room temperature. Primary antibodies in blocking solution were added and incubated overnight at 4 °C. The primary antibodies used for the immunolabeling are presented in Supplementary Table 1. After rinsing, samples were incubated with donkey Alexa Fluor-conjugated secondary antibodies (1:500; Invitrogen) for 1 h at room temperature. Nuclear counterstaining was carried out with 4′,6-diamidino-2-phenylindole (DAPI). Images were captured by a confocal microscope (LSM 800 or 900, Zeiss) using 20× or 40× (oil) objective. Marker-positive and total cells (DAPI) were quantified using ImageJ software combined with manual counting and an automated cell counting platform (ImageJ with automated thresholding).

### qPCR with reverse transcription

Total RNA was isolated from the culture using the RNeasy mini kit (Takara) according to the manufacturer’s protocol and quantified with NanoDrop. For cDNA synthesis, the reverse transcription reaction was carried out with Prime Script TM Master Mix (Takara), and quantitative PCR (qPCR) was performed using primers specific for the genes of interest (presented in Supplementary Table 2) with SYBR Premix Ex Taq II (Tli Rnase H Plus; Clontech) in 20-μl reaction volumes. We quantified relative gene expression levels using the${2}^{\varDelta \varDelta \mathrm{Ct}}$ method, using β-actin as an internal control for normalization.

### Electrophysiology

Whole-cell current clamp or voltage clamp experiments were conducted on an EPC-10 amplifier (EPC-10 USB; Heka) using PatchMaster software (Heka). Two-component capacitive compensation was optimized at rest, and series resistance was compensated to 50%. Analog data were filtered at 2 kHz and digitized at 10 kHz or 50 kHz. For current clamp, standard pipette solution contained (in mM) 115 K-gluconate, 15 KCl, 10 KOH, 5 MgCl_2_, 0.1 CaCl_2_, 5 Na_2_ATP, 0.5 NaGTP, 0.5 Na–cGMP, 0.5 cAMP, 1 BAPTA, 10 HEPES and 50 sucrose (pH adjusted with KOH to 7.2, osmolarity 320–330 mOsm) and the standard extracellular solution contained 140 NaCl, 5 NaOH, 5 KCl, 2 CaCl_2_, 5 MgCl_2_, 15 sucrose, 15 HEPES and 25 dextrose (pH adjusted with NaOH to 7.3, osmolarity 330–340 mOsm). We injected a series of step currents with incremental amplitude (six steps, with five Picoampere (pA) increments) to elicit action potentials (APs).

The spontaneous inhibitory postsynaptic potentials were recorded under −60 mV. Voltage-clamp recordings were performed in whole-cell configuration with internal solution containing (in mM) 135 CsCl_2_, 10 Hepes, 1 EGTA, 1 Na–GTP and 1 QX-314 (pH adjusted to 7.4, 310 mOsm), whereas current-clamp internal solution contained (in mM) 130 KMeSO_3_, 10 NaCl, 10 HEPES, 2 MgCl_2_, 0.5 EGTA, 0.16 CaCl_2_, 4 Na_2_ATP, 0.4 NaGTP and 14 Tris–creatine phosphate (pH adjusted with KOH to 7.3, 310 mOsm). The bath solution contained (in mM) 140 NaCl, 5 KCl, 2 CaCl_2_, 1 MgCl_2_, 10 glucose and 10 HEPES–NaOH (pH 7.4).

### Bulk RNA sequencing analysis

Raw FASTQ files were first processed using fastp (v1.0.1) with default settings; thereafter, transcript quantification was performed using Salmon (v1.10.1) in mapping-based mode with default parameters and, finally, differential expression analysis was carried out in DESeq2 (v1.46.0). Per bulk RNA sequencing data, iGABAPs have 3552 significantly upregulated genes (log_2_ fold change >1 and Benjamini–Hochberg-adjusted *P* value <0.05) and 2690 significantly downregulated genes (log_2_ fold change <−1 and Benjamini–Hochberg-adjusted *P* value <0.05) compared with dorsal spinal neural progenitors (DSNPs).

### Animals

We used Sprague–Dawley male rats (230–260 g) from the Centre for Comparative Medicine Research, Li Ka Shing Faculty of Medicine, The University of Hong Kong. All animal protocols were approved by the Committee on the Use of Live Animals in Teaching and Research of The University of Hong Kong. Health guidelines for laboratory animal care and safety were strictly followed. Animals had free access to food and water throughout the study.

### Contusion moderate SCI model

All surgical procedures were performed under anesthesia (75–100 mg/kg ketamine, 10 mg/kg xylazine) via intraperitoneal injection. As described^19^, We conducted a moderate thoracic (T9–T10) traumatic SCI in rats using the NYU-MASCIS weight‑drop impactor with a 10 g rod featuring a flat circular tip. After performing the spinal contusion, muscle and skin layers were sutured with 4.0 polyglactin. The bladder of each injured animal was squeezed manually twice a day after SCI for 2–3 weeks. Antibiotics Baytril (enrofloxacin, 10 mg/kg, intramuscular) and analgesics, including buprenorphine (0.05 mg/kg) and meloxicam (2 mg/kg) were administered for 7 days after injury.

### Cell engraftment

A total of 10 days after SCI surgery, animals underwent a second procedure for cell implantation. The rats were anesthetized as described above. The original incision was reopened, and the injury sites were re-exposed. Medium, DSNPs and TF-iGABAPs were resuspended in DMEM/F12 medium supplemented with 10 µM Rock inhibitor at a density of 10^5^ cells/µl without growth factors. Cells were kept on ice throughout the procedure. Two injections were performed at the injury site, each delivering 2.0 µl of the cell suspension at −0.5 and +0.5 mm distance from the dorsal middle line (0 mm). The injection was performed using a 30 G syringe (Hamilton) connected with a micropump (RWD), with the animals tightly fixed in a stereotaxic apparatus (RWD). The injection rate was 250 nl/min. The syringe was left in place for an additional 10 min before and after the injections. At the end of the procedure, the muscle and skin layers were sutured with 4.0 polyglactin, and the animals received subcutaneous injections of buprenorphine (0.03 mg/kg) and meloxicam (2 mg/kg) for the first 3 days after surgery and oral administration of enrofloxacin (2.5%) for the first 7 days after surgery. Cyclosporine (5 mg/kg) was subcutaneously injected every day for immunosuppression until death. Animals underwent functional testing for up to 12 weeks and were killed for anatomical analysis by transcardial perfusion with 4% formaldehyde.

### Injection of viruses for anterograde transneuronal labeling

A total of 3 weeks before death, the skulls of anesthetized rats (ketamine (80 mg/kg) and xylazine (10 mg/kg)) were tightly fixed to a stereotaxic apparatus (RWD). rAAV-hSyn-CRE-WPRE-hGH pA + AAV-DIO-mCherry (brainVTA, 2.5 × 10^12^ vg/ml) was injected into five spots of the right motor cortex. A vertical midline incision was made from between the eyes to the posterior skull. The injection area on the right hemisphere was defined in a rectangle measuring 2 mm (from 1.0 mm anterior to −1.0 mm posterior to the bregma) by 1.5 mm (lateral to the bregma). A drill (0.6 mm) was used to create the injection sites on the skull. Injections were performed using a 33 G syringe (Hamilton) attached to a micropump (RWD). Each injection delivered 0.5 µl of the virus solution into the motor cortex at a rate of 100 nl/min. The injector tip was left in place for additional 5 min before and after the injections. Animals were killed 3 weeks after injection, and postsynaptic structures were examined for the presence of cell body labeling.

### Immunofluorescence

Animals were sacrificed by intraperitoneal injection of overdose pentobarbitone (150–200 mg/kg) before, followed by perfusing with 0.9% saline and 4% paraformaldehyde. Spinal cords were removed, post-fixed, and sectioned at 20-μm intervals. Sections were dried overnight at room temperature. Sections were then incubated with primary antibodies overnight (Supplementary Table 1) followed by incubation with Alexa Fluor 488, 594 or 647-conjugated donkey secondary antibodies (1:500; Invitrogen) for 1 h at room temperature. For nuclear staining, DAPI was added to the final wash. Images were captured under confocal microscopy (LSM 800 or 900, Zeiss) using 10×, 20× or 40× (oil) objective using *Z*-stack or tiles. All primary and secondary antibodies are presented in Supplementary Table 1.

### Quantification of mCherry-labeled cells/axons or neural cell type in grafts

For quantification of neural differentiation or growth in human cell grafts, eight to nine randomly selected fields of grafts from six to eight animals per group were visualized using a Carl Zeiss LSM 800 or 900 confocal microscope at a magnification of 100× or 200×. The number of mCherry-expressing axons regenerating into grafts in the lesion sites was quantified as previously described^13,20^. In brief, using ZEN offline, dorsal-to-ventral virtual lines of one in six sections (30-µm thickness) were placed at regular distances under 100× magnification and graft–host interface and the number of axons intercepting labeled for 5-HT or mCherry-labeled axons were examined and counted under 200× magnification.

### Behavioral studies for locomotor activity

All behavioral tests were performed between 12:00 and 18:00 in a room specialized for animal behavioral tests, and the light and temperature were controlled by experimenters who were blinded to the experiment. Rats were allowed to adapt to the environment for 30 min before testing. Two investigators blinded to group identity assessed the outcomes.

For sensation, the mechanical nociceptive threshold was assessed using the von Frey test as described previously. In brief, rats were placed individually in transparent plastic boxes on a stainless steel mesh-bottomed platform for 30 min to acclimate to the environment. Hind paw withdrawal threshold (PWT), which responded to blunt von Frey filaments connected to a calibrated electronic von Frey filament esthesiometer (IITC Life Science), was recorded automatically by the esthesiometer. The filaments were applied perpendicularly to the plantar surface of the ipsilateral hind paw. Quick withdrawal (but not due to locomotion) or licking of the paw was considered a positive response. Three repeated measurements were made per animal under each test session with a 5-min interval.

Cold and hot avoidance was assayed by two temperature preference tests (Ugo Basile). Rats were allowed to acclimate on the plate at ambient temperature (30 °C ± 2 °C) for 60 min. At the end of the habituation period and 2 h before ‘warm-up’, the temperature-controlled plates were turned on at two different temperatures, with one side of the plate set to 30 °C ± 1 °C for cold avoidance or 45 °C ± 1 °C for hot avoidance and the other side remained at an ambient temperature (30 °C ± 2 °C). The locomotor activity of the rat was recorded by a portable digital camera and analyzed by a Smart 3.0 video tracking software. Given the learning and memory ability of rats, the temperature settings between the reference plate and test plate were interchanged for the next trial. Each rat was tested twice for each set of parameters, and rats were re-habituated between trials.

For locomotion activity assay, the Basso, Beattie and Bresnahan (BBB) open-field 21-point locomotion rating scale was used in the weekly assessments of rats conducted by two independent observers^21^. Grid walk assessment was performed using a modified grid (4 cm × 6 cm grids), and hind limb foot drops were recorded as a measure of hind limb sensorimotor function. Two investigators blinded to group identity assessed the outcomes. Foot drops were recorded if the rat was unable to grasp a grid rung with a hind paw during stepping and paw placement, resulting in a foot drop below the grid. The percentage of total paw replacements = total steps – foot drops / total steps (including left and right hind limb). Testing was performed on uninjured rats before surgery as a baseline measurement and then every 2–3 weeks after injury. Three trials per rat were evaluated, and the scores were averaged for the analysis. For the foot fault scoring, a qualitative analysis was performed on skilled walking as previously described^22^. In brief, the score was defined as follows: correct placement 6 points; partial placement 5 points, placement correction 4 points, replacement 3 points, slight slip 2 points, deep slip 1 point and total miss 0 points. With the limb that started the walk, consecutive steps were then estimated. Three trials per rat were evaluated, and the scores were averaged for the analysis.

### Statistical analysis

For comparison between two groups, a two-tailed unpaired Student’s *t*-test was used at a designated significance level of *P* < 0.05. The normality assumption (verified using the Shapiro–Wilk test, all *W* > 0.9, *P* > 0.05) and the homogeneity of variances assumption (verified by Levene’s test, all *P* > 0.05) were confirmed for the use of parametric tests, justifying the use of parametric tests. The measurements taken at different groups or time points were compared using one-way analysis of variance (ANOVA) followed by Tukey’s multiple comparisons or by two-way repeated-measures ANOVA with Bonferroni’s post hoc test. For all tests, values were considered significantly different at *P* < 0.05. ^*^P < 0.05, ^**^P < 0.01, ^***^P < 0.001, ^****^P < 0.0001. Gene expression data were analysed using two-tailed one-sample Student’s *t*-tests when compared with the baseline control group. Statistical analyses were performed using Prism 8 (Graphpad Software). All data were presented as mean ± s.e.m. The statistical details of each experiment can be found in the figure legends. No statistical methods were used to calculate sample size estimates, but sample sizes in the current study were similar to previous reports^12,13,23,24^.

## Results

### Traumatic SCI induced CNP and impaired locomotor activity

**Fig. 1:**
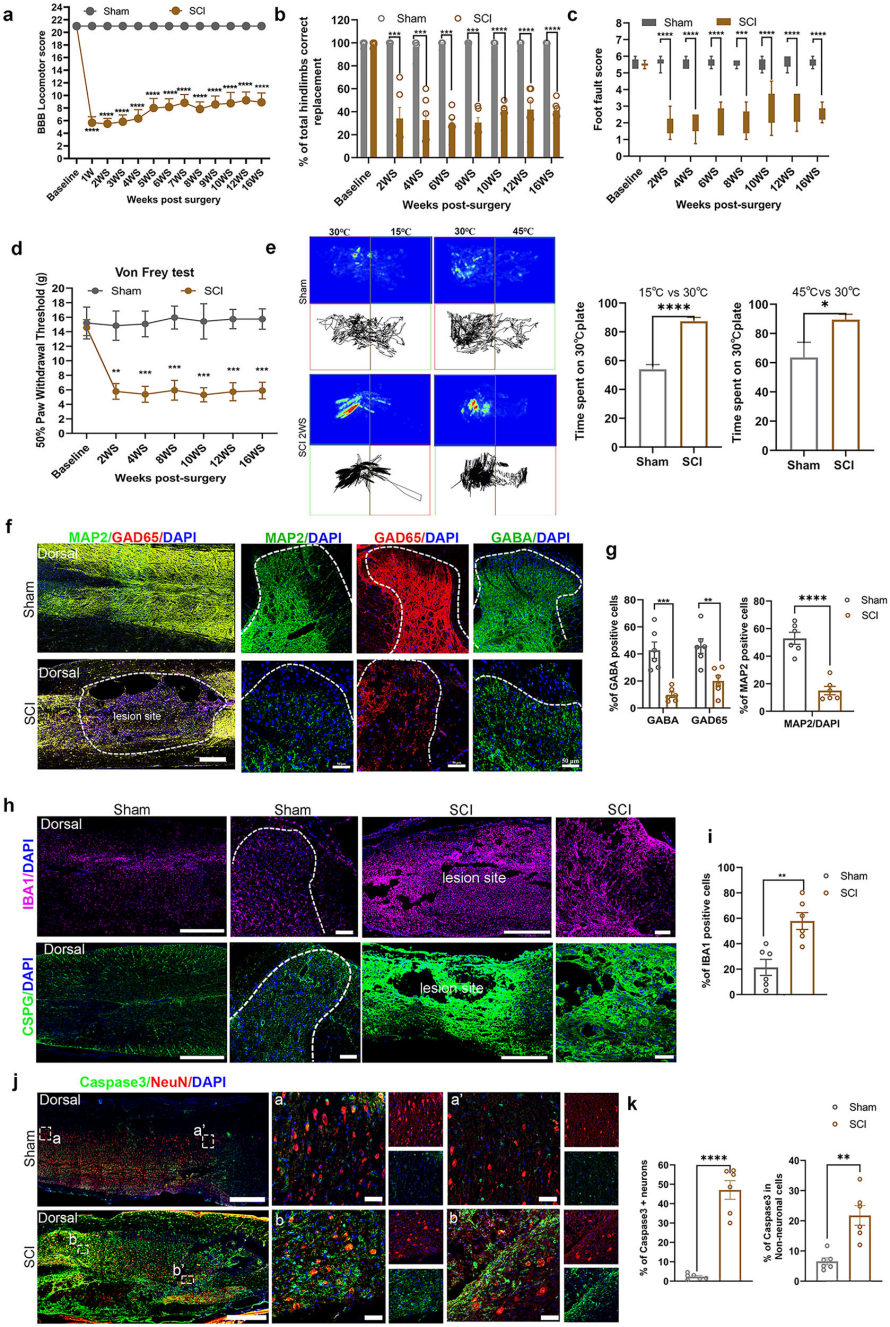
Moderate traumatic SCI induced GABA deficiency and CNP. **a** BBB locomotor scores in sham and SCI groups before and after injury. Two-way repeated-measures ANOVA with Bonferroni’s post hoc test. **b** Quantitative analysis of hind limb placement accuracy in the grid walk test in sham and SCI rats. Two-tailed unpaired *t*-test. **c** Foot fault score analysis of hind limb measured by rating scale for foot placement in the skilled ladder rung walking test (correct placement 6 points, partial placement 5 points, placement correction 4 points, replacement 3 points, slight slip 2 points, deep slip 1 point and total miss 0 points). Two-tailed unpaired *t*-test. **d** Time course of the mechanical allodynia, as measured by the von Frey force threshold for withdrawal in sham and SCI rats. Two-way repeated-measures ANOVA with Bonferroni’s post hoc test. **e** Representative track imaging of the two-plate preference test at 30 °C versus 15 °C or 30 °C versus 45 °C after SCI (2 weeks after injury). Right: occupancy quantification of 30 °C versus 15 °C or 30 °C versus 45 °C after 2 weeks of injury. Two-tailed unpaired *t*-test. **f** Representative immunofluorescence images for MAP2, GAD65 or GABA in sagittal sections and transverse sections of dorsal view in sham and SCI rats at 2 weeks after injury. Scale bar in sagittal view, 200 µm; scale bar in transverse sections, 50 µm. **g** Quantification of indicated markers in **f**. Two-tailed unpaired *t*-test. **h** Representative immunofluorescence images for Iba1 and CSPG in spinal cord sagittal sections and transverse sections in sham and SCI rats at 2 weeks after injury. Scale bar in sagittal view, 200 µm; scale bar in transverse sections: 50 µm. The lesion cavity is formed with surrounding dense CSPG immunoreactivity. **i** Quantification of the percentage of IBA1/DAPI and the relative intensity of CSPG in **h** two-tailed unpaired *t*-test. **j** Representative immunofluorescence images for caspase-3, NeuN and DAPI in sham and SCI rats at 2 weeks after injury. a–a′ and b–b′ show higher magnification of caspase-3 and NeuN. Scale bar in sagittal view, 200 µm; scale bar in a–a′ and b–b′, 50 µm. **k** Quantification of the proportion of caspase-3 in NeN^+^ and NeuN^−^ cells. Two-tailed unpaired *t*-test. All data are expressed as mean ± s.e.m. ^*^P < 0.05, ^**^P < 0.01,^***^P < 0.001, ****P < 0.0001.

To recapture clinical phenotypes observed in patients with SCI with CNP development, we have established a moderate traumatic SCI model at the thoracic (T9–10) level^25^, which induced a relatively mild loss of hind limb locomotor functions and regained weight-bearing plantar stepping in one or two hind paws by 2–3 weeks after injury (Fig. 1a–c). These SCI rats started to develop neuropathic pain from 2 weeks after injury, as compared with sham groups, which were characterized by mechanical allodynia and thermal hypersensitivity (Fig. 1d,e). Immunofluorescence analysis of the longitudinal (dorsal view) and cross spinal cord showed a significant reduction in GABAergic inhibitory neurons, marked by GAD65 and MAP2, as well as decreased GABA expression levels 2 weeks after injury (Fig. 1f,g), coinciding with the development of CNP. In addition, we detected a massive amount of activated microglia (IBA1) accumulating around and within the lesion sites, which are known to produce proinflammatory cytokines, reactive oxygen species and proteases, contributing to secondary neuronal injury during the acute-to-chronic stage and other complications^26,27^. Chondroitin sulfate proteoglycans (CSPG) produced by the glial scar were found to be highly enriched around the cavity, creating an inhibitory barrier that prevents neuronal differentiation and axonal regeneration (Fig. 1h,i). In response to the detrimental microenvironment, we observed extensive apoptosis of spinal cells, marked by caspase-3, including neurons (NeuN^+^) and non-neuronal cells, after 2 weeks of injury (Fig. 1j,k). Altogether, the SCI causes a dramatic loss of GABAergic inhibitory neurons in the dorsal spinal cord, which is highly associated with CNP development.

### Validating key TFs in human dorsal spinal cord GABAergic neurons derived from human PS cells

**Fig. 2:**
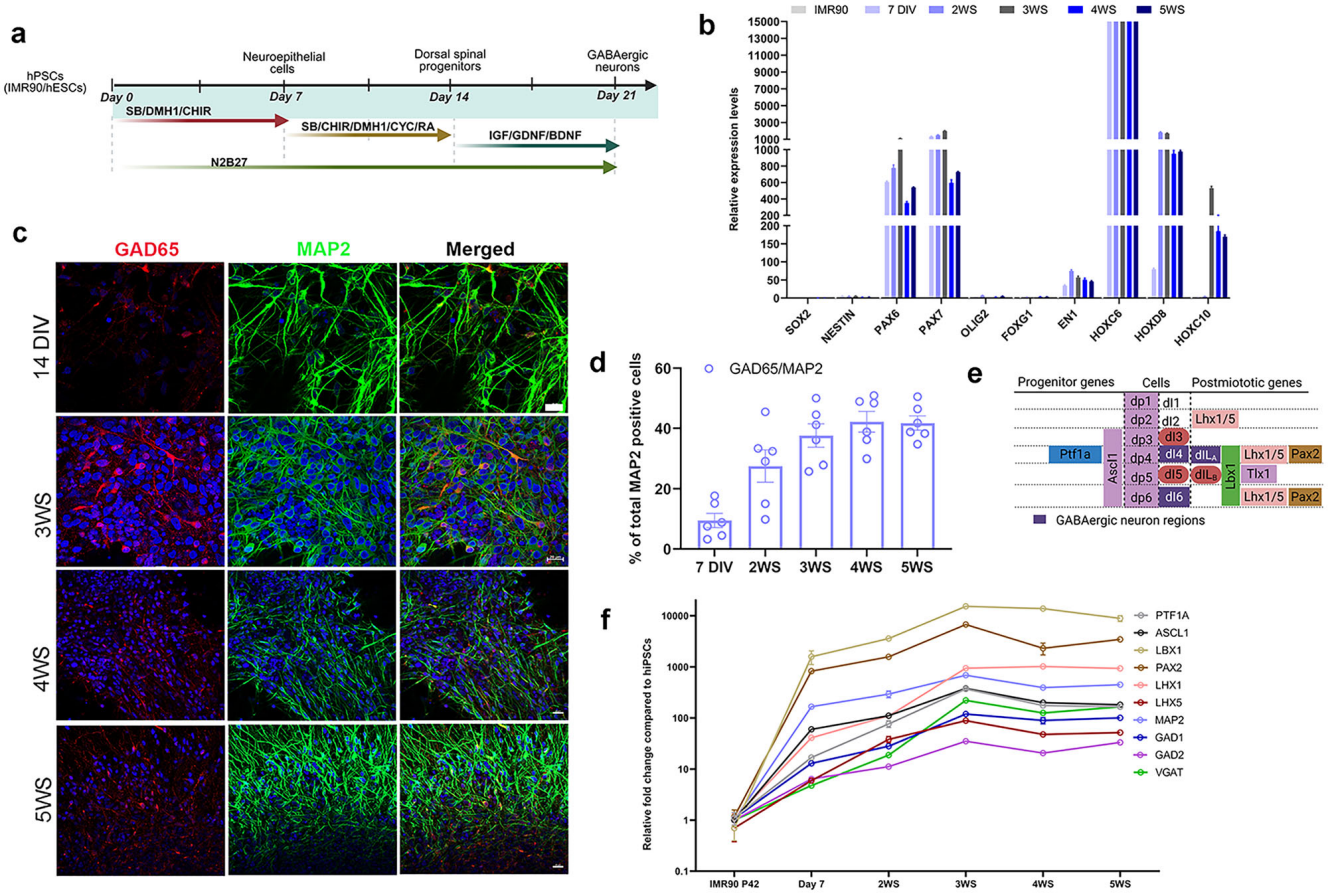
Validating TFs for dorsal spinal GABAergic neurons specifications in a human context. **a** Experimental strategy for generating dorsal spinal interneurons from human PS cells using small molecule induction. **b** Time course qPCR analyses showing expression levels of indicated spinal, dorsal and neural markers, with either low or no expression of ventral and brain markers in neural progenitors compared with human PS cells. **c** Immunofluorescence of MAP2 and GAD65 from 14 days to 5 weeks of neuronal differentiation. DAPI was used to stain DNA in nuclei. **d** Quantification of the percentage of GAD65 in MAP2^+^ cells from 7 days to 5 weeks of neuronal differentiation. **e** Schematic diagram showing key TF expression for GABAergic neuronal specification in the developing neural tube. **f** qPCR analyses of indicated gene expression levels at different time points during neuronal differentiation. The levels of each transcript were normalized to human PS cells. Each bar represents the mean ± s.e.m. of three independent experiments. DIV, days in vitro; WS, weeks in vitro.

Although several studies have successfully generated human GABAergic neurons in various regions of the brain, it is noted that brain and dorsal spinal GABAergic neurons originate from distinct developmental pathways and are regulated by different transcriptional and signaling mechanisms^15,19,28^. In addition, key factors in specifying dorsal spinal cord GABAergic identity are less validated in a human context. To address this, we first generated dorsal spinal cord neural progenitors derived from human PS cells and then differentiated these progenitors into interneurons using defined small molecules, which should contain a subpopulation of GABAergic neurons (Fig. 2a). The neural progenitors were characterized as dorsal spinal cord identity, as indicated by the increased expressions of thoracic spinal markers (*HOXC6*, *HOXC8* and *HOXC10*) and dorsal neural markers (*PAX3*, *BRN2*, *SOX2*, *Nestin* and *PAX6/7*) during neural induction. Importantly, we did not detect the induction of ventral or brain patterning markers, including *OLIG2, FOXG1* and *EN1*^13,29^ (Fig. 2b). These dorsal spinal progenitors generate a mixture of subtypes of neurons and less than 40% differentiated neurons expressing GABAergic neuronal markers, GAD65, after 5 weeks of neuronal differentiation, indicating the heterogeneous differentiated neurons using small molecules (Fig. 2c,d). Previous studies in animal models have identified *Ptf1a* as a key factor in specifying the dorsal spinal GABAergic neural progenitor domain (dP4), along with a few TFs that promote and are specifically expressed in post-mitotic GABAergic neurons, including *Lbx1*, Pax2 and Lhx1/5^30–34^ (Fig. 2e). We further examined whether expression of these TFs were positively correlated with human GABAergic neuronal fate determination and differentiation, which might underlie their potency to directly convert human PS cells into functional GABAergic neurons. Our qPCR analysis identified an initial significant upregulation of proneural genes *PTF1A* and *ASCL1* during the GABAergic progenitor stage from 7 to 21 days of neuronal differentiation (Fig. 2f). We also detected a gradual elevation of mature GABAergic neuronal markers, *GAD1*, *GAD2* and *VGAT*, correlating the expressions of a TFs set, *PAX2*, *LHX1/5* and *LBX1* (Fig. 2f). In summary, we identified that the expression of regional and lineage-specific TFs associated with the determination of dorsal spinal GABAergic neuronal fate was conserved between chicken, mouse and human, suggesting that a combination of TFs may be critical for dorsal spinal GABAergic lineage reprogramming.

### Identifying TF combinations for inducing human dorsal spinal cord GABAergic neurons

**Fig. 3:**
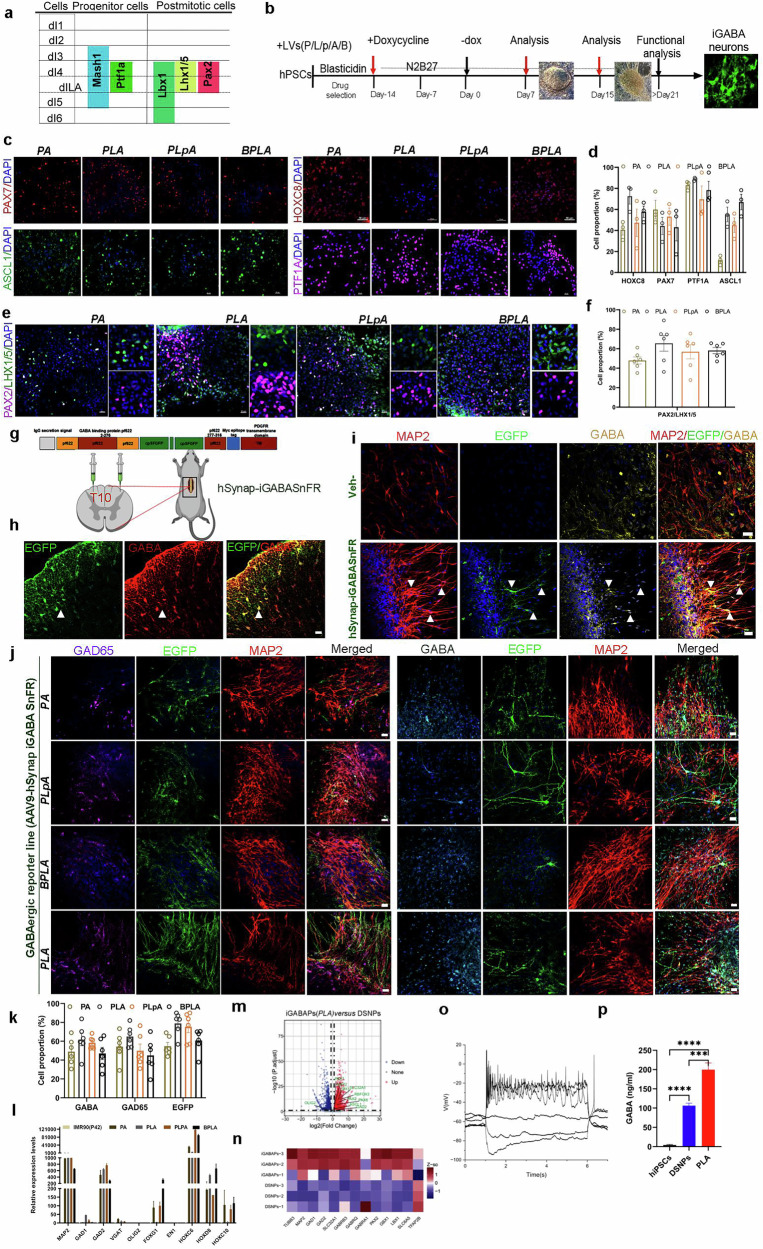
Screening TFs for rapidly generating dorsal spinal GABAergic neurons. **a** Schematic of selected TF expressions for the direct generation of GABAergic neurons. **b** Experimental strategy for generating the dorsal spinal cord using selected TFs. **c** Immunofluorescence of PAX7, HOXC8, PTF1A and ASCL1 in human PS cells overexpressing different TF combinations (*PA*, *PLA*, *PLpA* and *BPLA)* at 1 week after dox withdrawal. TF abbreviations: *PTF1A (P)*, *PAX2 (p)*, *LBX1 (L), ASCL1 (A)* and *BRN2(B)*. DAPI was used to stain DNA in nuclei. **d** Quantification of the percentage of indicated markers as shown in **c**. **e** Immunofluorescence of PAX2 and LHX1/5 in human PS cells with different overexpression of TFs: *PA*, *PLA*, *PLpA* and *BPLA* at 2 weeks after dox withdrawal. DAPI was used to stain DNA in nuclei. **f** Quantification of the percentage of indicated markers as shown in **e**. **g** Schematic of intraspinal injection strategy for GABAergic reporter (AAV-hSynap-iGABASnFR) in the dorsal horn. **h** Representative immunofluorescence images for EGFP (hSynap-iGABASnFR), MAP2 and GABA in the dorsal spinal cord at 2 weeks after injection. Scale bar, 20 µm. **i** Immunofluorescence of EGFP (hSynap-iGABASnFR) and GABA at 5 weeks of neuronal differentiation from human PS cells. Veh is a negative control without AAV-hSynap-iGABASnFR transduction. DAPI was used to stain DNA in nuclei. Scale bar, 20 µm. **j** Representative immunofluorescence images for GFP (hSynap-iGABASnFR), MAP2 and GABAergic neuronal markers GAD65 and GABA in human PS cells with different combinations of TF overexpression (PA, PLA, PLpA and BPLA) at 2 weeks after dox withdrawal. Scale bar, 20 μm. **k** Quantification of the percentage of indicated markers in **j**. **l** qPCR analysis of markers associated with GABAergic neuronal differentiation and maturation in human PS cells with different combinations of TF overexpression, PA, PLA, PLpA and BPLA at day 21. One-way ANOVA followed by Tukey’s post hoc test. **m** Volcano plot highlighting differentially expressed genes (DEGs; false discovery rate (FDR) <0.05; red, upregulated; blue, downregulated) in iGABAPs (PLA induced) compared with DSNPs. **n** Heat map of DEGs associated with GABAergic neuronal fates (red, upregulated; blue, downregulated) in iGABAPs (PLA induced) compared with DSNPs. **o** Repetitive series of APs in response to step-current injections in PLA-induced GABAergic neurons. **p** Quantification of GABA production ELISA from the indicated groups. One-way ANOVA followed by Tukey’s multiple comparison test. ^***^P < 0.001, ^****^P < 0.0001. Three independent experiments. All data are expressed as mean ± s.e.m.

To identify TF combinations capable of directing human PS cells into dorsal spinal cord GABAergic neuronal lineages effectively, we initiated our screen with PTF1A owing to its essential role in specifying dorsal spinal GABAergic progenitor identity and inducing various GABAergic targets. We subsequently incorporated LBX1 for its crucial role in the maturation phase, where it functions with PTF1A (P) to induce key post-mitotic determinants such as PAX2 and LHX1/5^30–34^. PAX2 (p) was selected owing to its maintained expression pattern in mature GABAergic neurons in adults^35^ (Figs. 2f and 3a). We also included ASCL1 (A) or BRN2 (B) to enhance the overall neurogenic efficiency^29,36,37^. We systematically tested the efficacy of various TF combinations—*P (PTF1A)*, *PA (PTF1A* + *ASCL1)*, *PLA (PTF1A* + *LBX1* + *ASCL1)*, *PLpA (PTF1A* + *LBX1* + *PAX2* + *ASCL1)* and *BPLA (BRN2* + *PTF1A* + *LBX1* + *ASCL1)*—in converting human PS cells toward a dorsal spinal GABAergic neuronal fate. For this purpose, we generated monocistronic dox-inducible lentiviral vectors containing *PAX2, ASCL1, LBX1, PTF1A* or *BRN2* and transduced human PS cells with the selected TF combinations in the presence of dox for 2 weeks (Fig. 3b). Initial observations revealed dramatic formation of neural-like cells at day 7 after dox withdrawal (−dox day 7) across all TF combinations (Supplementary Fig. 1a). These cells showed robust neurite outgrowth, particularly *PLA*, at day 14 after Dox withdrawal (−dox day 14) (Supplementary Fig. 1b). Immunostaining demonstrated that cells transduced with *P*, *PA*, *PLA*, *PLpA* or *BPLA* highly expressed markers of dorsal thoracic identity (PAX7 and HOXC8) and GABAergic progenitor (PTF1A and ASCL1) (Fig. 3c,d and Supplementary Fig. 1c) by day 7 after dox withdrawal. Importantly, these TF combinations induced accelerated differentiation progression, expressing a high percentage of mature GABAergic neuronal markers, PAX2 and LHX1/5, by day 14 after dox withdrawal (Fig. 3e,f and Supplementary Fig. 1d,e). Notably, the *PLA* combinations exhibited the highest efficacy in converting human PS cells into dorsal spinal GABAergic neurons (Fig. 3e,f and Supplementary Fig. 1d,e).

To assess the functional properties of these induced GABAergic neurons, we used an intensity-based GABA-sensing fluorescence reporter (iGABASnFR-GFP)^38^, driven by the Synapsin promoter, to detect GABA release. This reporter was validated in vivo via intraspinal injection into the spinal cord of rats and in vitro by transfecting interneurons derived from human PS cells after 5 weeks of neuronal differentiation, where it colocalized with dorsal GABAergic neurons marked by MAP2 and GABA (Fig. 3g–i). By day 14 after dox withdrawal, we observed that all TF combinations induced iGABASnFR-GFP expression, co-expressing with GABAergic markers (GAD65, GABA and MAP2), among which *PLA* induced the highest GFP expression (Fig. 3j,k). Consistent with these findings, qPCR analysis confirmed robust induction of markers for spinal cord identity (HOXB1, *HOXC4*, *HOXC6*, *HOXD8* and *HOXC10*), dorsal neural (*PAX6/7*) and mature GABAergic neurons (*GAD1/2*, *LBX1* and *PAX2*) without expression of markers for ventral (*OLIG2*) or anterior brain identify (*OTX1* and *EN1*) during TF reprogramming (Fig. 3l and Supplementary Fig. 1f). These findings demonstrate the ability of the selected TF combinations to efficiently generate functional GABAergic neurons with dorsal spinal cord identity and confirm that *PLA* is an effective combination for subsequent therapeutic evaluation in the SCI model.

We further characterized *PLA*-iGABAPs, compared with DSNPs derived from human PS cells, before grafting in the SCI model. iGABAPs demonstrate a significant upregulation of key markers associated with GABAergic differentiation and dorsal spinal identity compared with control DSNPs (Fig. 3m,n). In addition, whole-cell patch clamp recordings showed that the neurons derived from iGABAPs were able to fire repetitive APs with prominent spike adaptation on depolarization and they exhibited both active and passive membrane properties, indicating functional maturation (Fig. 3o). These induced GABAergic neurons also displayed spontaneous inhibitory postsynaptic potentials, which were inhibited by adding picrotoxin (PTX), channel blockers of GABA_A_ receptors, confirming the predominant formation of inhibitory synapses (Supplementary Fig. 1g). In addition, we performed biochemical quantification of GABA release using a GABA enzyme-linked immunosorbent assay (ELISA) kit (Fig. 3p), which shows dramatic elevations in GABA production compared with human PS cells and DSNPs.

### Induced GABAergic neurons modulated the injured niche and promoted survival and connectivity

To assess the therapeutic potential and efficacy of these induced GABAergic neurons in treating SCI and CNP, we transplanted GFP-expressing *PLA*-induced iGABAPs into a moderate SCI model with neuropathic pain. For comparison, we also grafted DSNPs previously derived from human PS cells. A total of 1 × 10^5^ cells were injected bilaterally into the injury site without embedding Matrigel gel-coated growth factors, and cyclosporine was administered daily to prevent immune rejection.

**Fig. 4:**
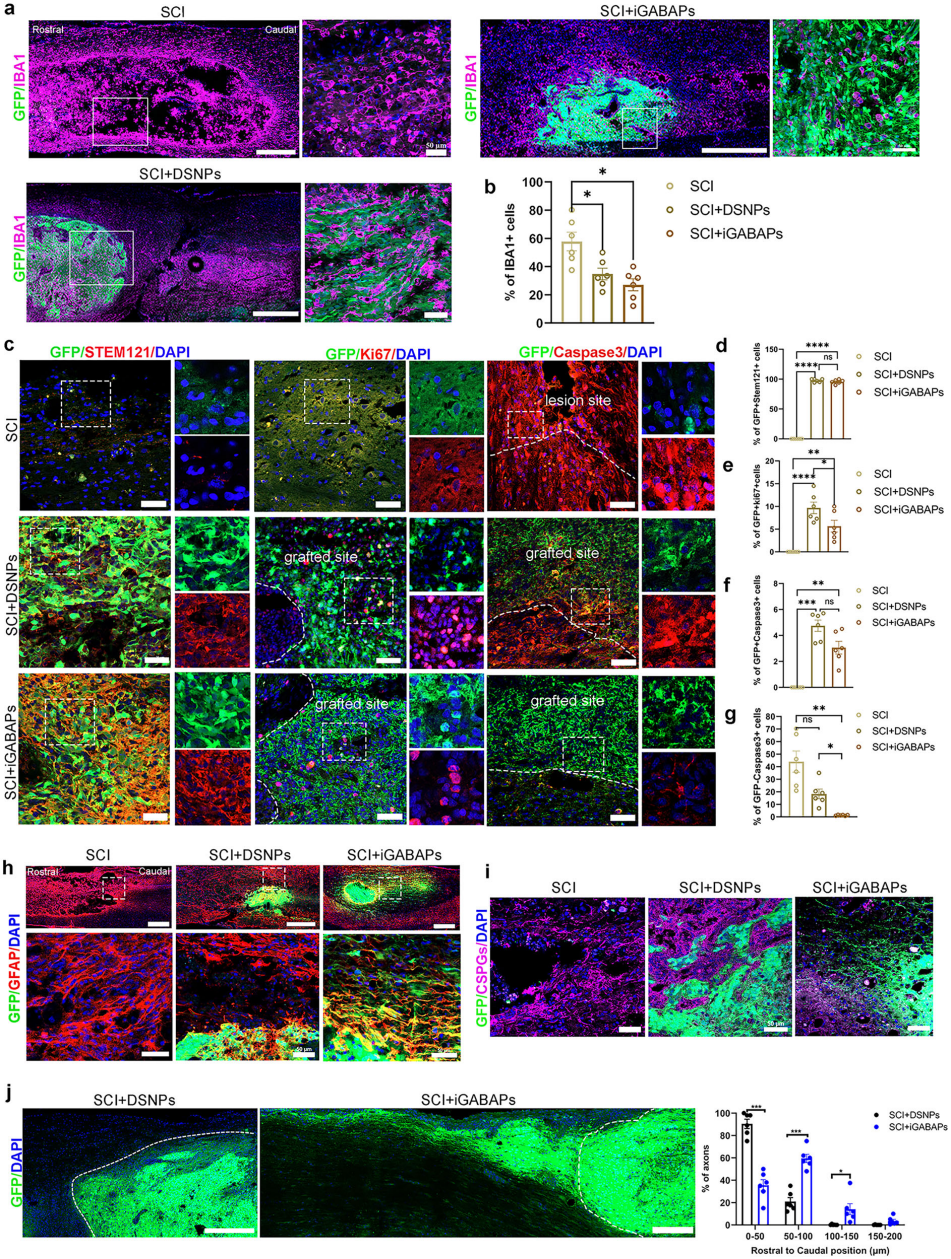
TFs induced GABAergic progenitor (iGABAPs), promote survival and modulate the injury niche. **a** Representative immunofluorescence images of GFP-labeled grafts costained with IBA1 in longitudinal sections of SCI, DSNP and iGABAPs grafts at 1 month after graft. Scale bar, 200 µm. The white box shows a magnified view with indicated markers. Scale bar, 50 µm. **b** Quantification of the percentage of IBA1-positive cells within lesion sites in SCI and SCI with DSNPs and iGABAPs grafts. One-way ANOVA with Tukey’s multiple comparison test. **c** Representative immunofluorescence images of GFP-labeled grafts costained with STEM121, Ki67 and Caspases 3 in transverse sections of DSNPs and iGABAPs grafts at 1 month after graft. The white box shows a magnified view with indicated markers. Scale bar, 50 µm. **d**–**f** Quantification of the percentage of STEM121 (**d**) Ki67 (**e**) and Caspases 3-positive cells (**f**) in SCI and SCI with DSNPs and iGABAPs grafts (GFP^+^). **g** Quantification of the percentage of Caspases 3-positive cells among non-graft cells (GFP^−^) in SCI and SCI with DSNPs and iGABAPs grafts. One-way ANOVA with Tukey’s multiple comparison test. **h** Representative immunofluorescence images for GFP and GFAP in spinal cord sagittal sections of SCI and SCI with DSNPs and iGABAPs grafts at 1 month after graft. Scale bar, 50 µm. The cystic lesion cavity formed with surrounding dense GFAP immunoreactivity (blue), whereas iGABAPs grafts (GFP-positive) crossed GFAP barriers. Bottom, magnified view with indicated markers. Scale bar, 50 µm. **i** Representative immunofluorescence images for GFP, CSPG and DAPI in spinal cord sagittal sections from SCI and SCI with DSNPs and iGABAPs grafts at 1 month after graft. The cystic lesion cavity formed with surrounding dense CSPG immunoreactivity. iGABAPs grafts with GFP expression attenuated CSPG graft–host interface. Scale bar, 50 µm. **j** GFP immunolabeling in spinal cord sagittal sections revealed that GFP-expressing iGABAPs grafts at injured sites generated robust axons extending into the host spinal cord caudally after 2 months following graft. Scale bar, 500 µm. Quantification of axon intercepts at specific distances from the graft–host border in the injured cord grafted with DSNPs and iGABAPs grafts, Two-tailed unpaired *t*-test. All data are expressed as mean ± s.e.m. At least three independent experiments. ^*^P < 0.05, ^**^P < 0.01, ^***^P < 0.001, ^****^P < 0.0001.

Functional recovery following cell transplantation depends on several key factors: graft survival, cell replacement and integration into the host tissue. The adult spinal cord niche after injury is challenging for transplanted cells to exert functions, which lacks neurotrophic support for neuronal maturation, and often favors astrocyte induction^11–13^. At 4 weeks after grafting, we observed that both grafted iGABAPs and DSNPs can survive and remain localized in the injured spinal cord. These grafts can moderate immune responses by reducing microglia activation (IBA1^+^) compared with nongrafted SCI controls (Fig. 4a,b and Supplementary Fig. 2). Although there were no significant differences in the proliferative rate (Ki67^+^; Fig. 4c–e) or apoptosis within the graft (Caspase-3^+^/GFP^+^; Fig. 4f) between groups, the iGABAPs treatment significantly reduced apoptosis in the host tissue. This was indicated by a markedly lower density of caspase-3+ non-GFP cells at the lesion periphery and within the graft site compared with the DSNPs group (Fig. 4g). CSPG, produced by reactive astrocytes at injury sites, function as a main inhibitory component to limit axonal outgrowth and regeneration, as well as oligodendrocyte replacement and remyelination^39^. Consistent with previous studies^40^, reactive astrocytes marked by GFAP and CSPG deposition created a chemical barrier that limited DSNPs outgrowth from the lesion site (Fig. 4h,i). Notably, levels of GFAP and CSPG near iGABAPs grafts were significantly reduced, enabling GFP-expressing axons to penetrate glial scars as early as 4 weeks after grafting (Fig. 4h,i). By 8 weeks after grafting, we observed a large number of GFP^+^ nerve fibers emerging from iGABAPs grafts into the uninjured spinal cord, extending much further caudally by more than 150 μm, whereas DSNPs grafts remained compact clusters with no axonal outgrowth extending through the lesion site or scar tissue (Fig. 4i,j). These findings indicate that iGABAPs grafts exhibited enhanced adaptation to the hostile injured environment, showing reduced apoptosis and robust axonal outgrowths. In addition, these iGABAPs grafts modulated the injured niche by reducing immune responses, promoting nongrafted cell survival and diminishing glial scar formation and inhibitory components, thereby facilitating neuronal reconnection.

### iGABAPs maturation and reciprocal synapse formation with host neurons

**Fig. 5:**
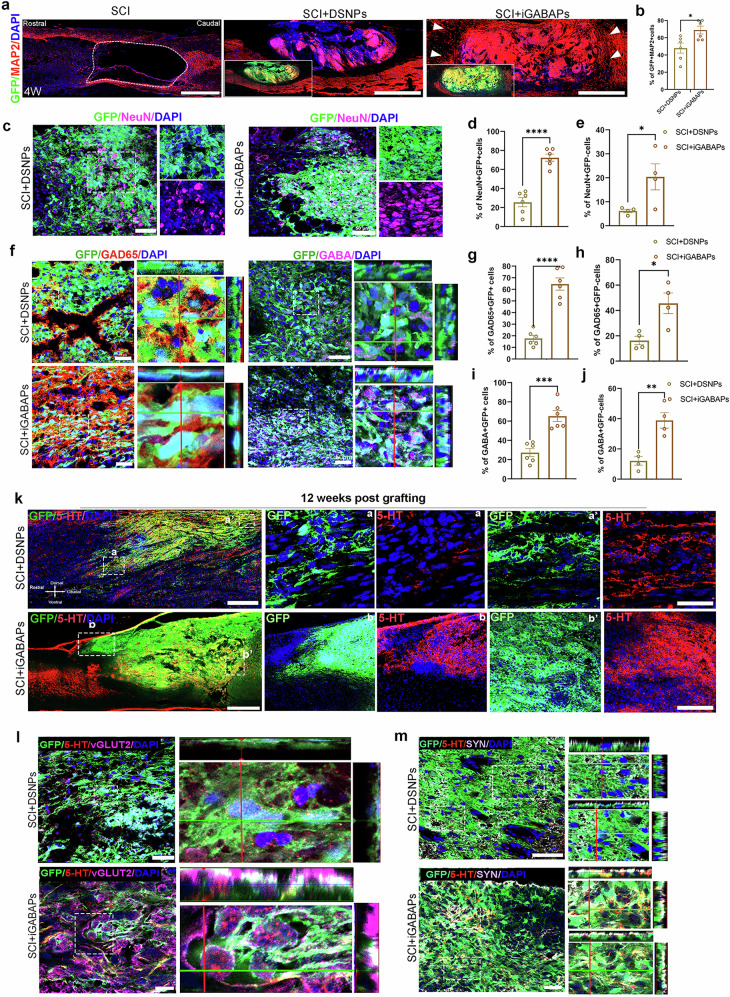
iGABAPs grafts effectively generate mature GABA for synaptic connection and promote endogenous neurogenesis. **a** Immunolabeling of GFP and MAP2 in sagittal spinal cord sections of SCI and SCI with DSNPs and iGABAPs grafts 1 month after grafting. Arrows highlight robust neurogenesis indicated by MAP2 signals crossing lesion sites both rostrally and caudally. **b** Quantification of the percentage of MAP2^+^ cells among GFP-labeled grafts in DSNPs and iGABAPs grafts. Student’s *t*-tests. **c** Spinal cord sagittal sections of SCI with DSNPs and iGABAPs grafts immunolabeled with NeuN antibody confirmed the differentiated neurons. Scale bar, 50 µm. **d**,**e** Quantification of the percentage of NeuN-positive cells in DSNPs and iGABAPs grafts (**d**) as well as adjacent host non-GFP cells (**e**). **f** Spinal cord sagittal sections of SCI with DSNPs and iGABAPs grafts immunolabeled with GAD65 and GABA. Scale bar, 50 µm. The white box shows a magnified view of *Z*-stack analysis for the colocalization of the indicated markers. **g**,**h** Quantification of the percentage of GAD65 (**g**) and GABA-positive cells (**h**) in DSNPs and iGABAPs grafts. **i**,**j** Quantification of the percentage of GAD65 (**i**) and GABA-positive cells (**j**) in adjacent host non-GFP cells from DSNPs and iGABAPs graft groups. (**k**) Representative images of host serotonergic axons immunolabeled with 5-HT (red) in DSNPs and iGABAPs grafts 3 months after graft. Scale bar, 100 µm. a–a′ shows higher magnification of 5-HT positive fibers innervating into the graft at different regions from rostral to caudal. Scale bar, 50 µm. **l** Triple immunolabeling of host 5-HT fibers, proximal VGLUT2 and GFP-terminals. The white box shows a zoomed-in view of *Z*-stack analysis for the colocalization of regenerating raphespinal axon with rat VGLUT2, suggesting synaptic connectivity in DSNPs and iGABAPs grafts. Scale bar, 50 µm. **m** Triple immunolabeling of host 5-HT fibers, proximal hsyn and GFP-terminals in DSNPs and iGABAPs grafts. The white box shows a zoomed-in view of *Z*-stack analysis for the colocalization, indicating the establishment of synaptic contacts between the host raphespinal fibers and grafted cells derived from iGABAPs. Scale bar, 50 µm.

We further examined the differentiation and maturation potency of iGABAPs grafts and their ability to establish synaptic connectivity with host neurons. At as early as 6 weeks, we observed a marked increase in the number and intensity of MAP2-positive neurons derived from iGABAPs grafts at the lesion site compared with the DSNPs grafts (Fig. 5a,b). In addition, MAP2 derived from iGABAPs grafts was not restricted within the lesion sites, which extended rostrally and caudally across the glial scar barrier, while MAP2 derived from DSNPs was more localized in the lesion sites (Fig. 5a,b). At this stage, iGABAPs already exhibited a substantial amount of postmitotic neuronal marker, NeuN (Fig. 5c,d), and mature GABAergic markers, GABA (Fig. 5f,g) and GAD65 (Fig. 5i,j). In line with previous studies showing prolonged maturation (>6 months) after grafting in SCI with predominantly adopted excitatory neuronal fates^13,23^, DSNPs grafts produced a small proportion of mature inhibitory GABAergic neurons at this time point (Fig. 5f–j). Strikingly, iGABAPs also promoted the expression of neuronal markers in host tissues through a non-cell autonomous manner. We detected a substantially increased number of NeuN (Fig. 5e), GABA (Fig. 5h) and GAD65 (Fig. 5k) expression from non-GFP cells around the iGABAPs graft. Compared with the DSNPs grafting group, the iGABAPs graft group exhibited a more robust descending serotonergic projection, marked by 5-HT^+^, which extended caudally over greater distances within lesion site^13,23,41^ (Fig. 5l). We also detected serotonergic axons that formed synaptic connections with grafted cell dendrites, and these contacts were marked by VGLUT2 from the host or the human presynaptic marker synaptophysin (hsyn), indicating effective establishment of host–graft connectivity (Fig. 5m). These colocalized synaptic contacts were barely detected in DSNPs grafts (Fig. 5m).

### iGABAPs graft effectively established the integration into host neural circuits

**Fig. 6:**
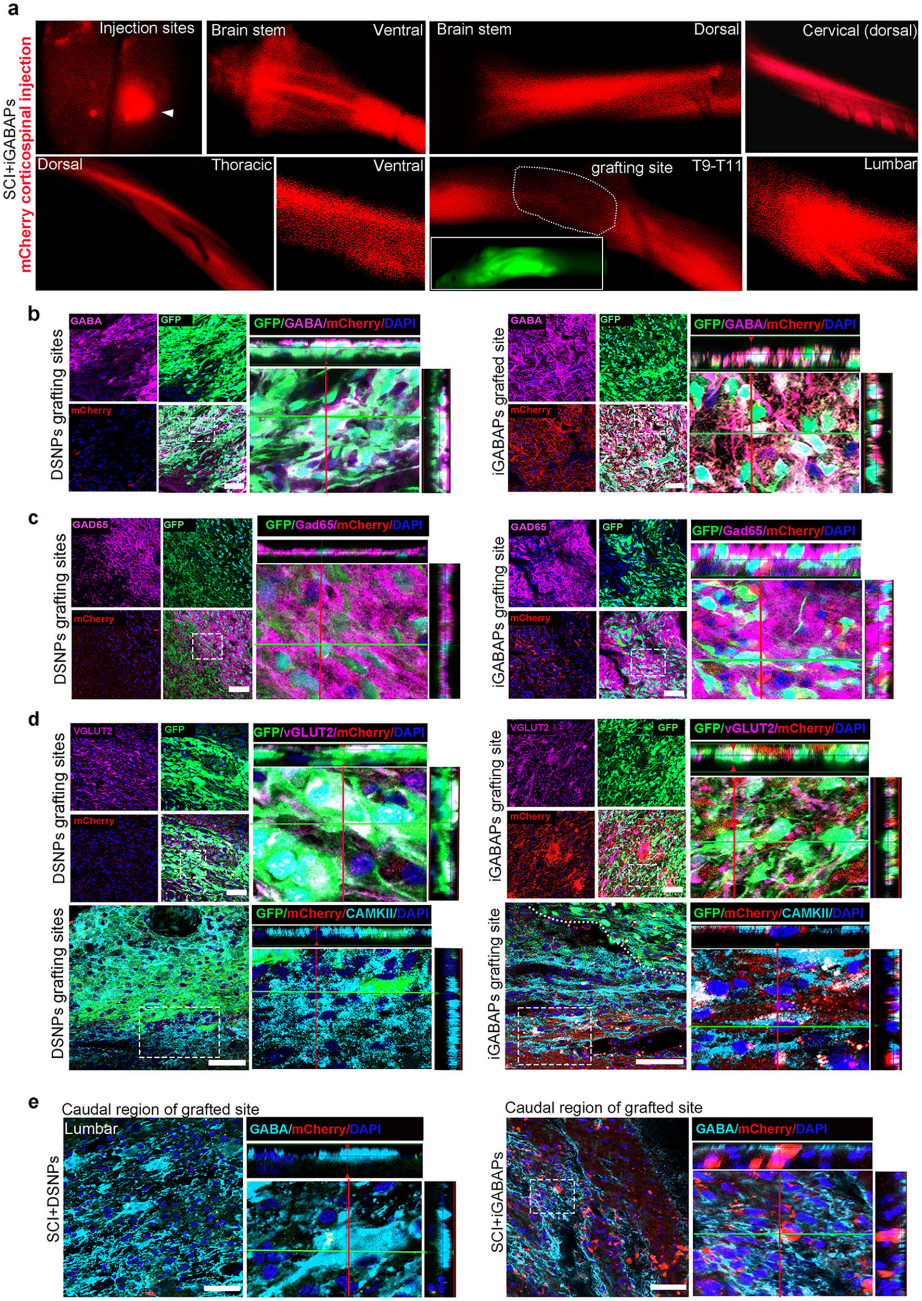
iGABAPs graft-initiated trans-synaptic AAV virus antegrade labeling of host connectivity. **a** Representative images showing mCherry^+^ in somatosensory and motor cortex (injection site) and trans-synaptic connection from the cortical region to the brainstem (ventral and dorsal view), cervical and thoracic region of the spinal cord, grafts at the lesion sites,caudal regions of the lesion and lumbar spinal cord. **b** Transverse sections labeled for mCherry, GFP and GABA/GAD65 at the grafting site of DSNPs and iGABAPs. Scale bars, 50 μm. The white box shows a magnified view of *Z*-stack analysis for the colocalization of indicated markers. **c** Triple labeling for GFP, mCherry-labeled corticospinal axons and rat VGLUT2 at the grafting site of DSNPs and iGABAPs groups. The white box shows a zoomed-in view of *Z*-stack analysis for the colocalization of the indicated markers. Scale bar, 50 μm. **d** Transverse sections labeled for mCherry, CaMKII and GFP at the grafting site of DSNPs and iGABAPs. Scale bars, 50 μm. The white box shows a zoomed-in view of *Z*-stack analysis for the colocalization of indicated markers. **e** Transverse sections of the lumbar spinal cord (caudal to the graft) labeled for mCherry and GABA (host inhibitory interneurons) from DSNPs and iGABAPs graft recipient. The white box shows a magnified view of *Z*-stack analysis for the colocalization of the indicated markers. Scale bars, 50 μm.

To assess functional integration of grafts with the host neural circuitry and re-establish synaptic connectivity over long distances, we performed anterograde viral tracing using rAAV-hSyn-Cre-WPRE-hGH-PA combined with AAV-DIO-mCherry^42^, injected into the somatosensory and motor cortex. At 3 weeks after injection, robust mCherry+ signals were detected in the injection sites (somatosensory/motor cortex) and in descending projections to the brainstemas well as cervical and thoracic spinal cord rostral to the injury in both iGABAPs grafts and DSNPs graft recipients (Fig. 6a and Supplementary Fig. 3). Notably, strong mCherry+ labeling extended caudally beyond the injury site in recipients with iGABAPs grafts, reaching thoracic and lumbar spinal regions, which indicates the restoration of trans-synaptic transmission from the somatosensory and motor cortical neurons to the lower spinal cord segments. By contrast, this caudal mCherry+ signal was markedly weaker in the DSNPs group. Immunofluorescence analysis within the graft site revealed that the robust mCherry signal in the iGABAPs group extensively overlapped with GFP+ grafted cells, which co-expressed the GABAergic neuronal markers GABA (Fig. 6b) and GAD65 (Fig. 6c), indicating the effective trans-synaptic spread from host neurons to the iGABPA grafts. Furthermore, we found that mCherry-labeled corticospinal axons regenerating into injury regions showed synaptic connections with host excitatory neurons (CaMKII^+^) and also exhibited bouton-like terminals that colocalized with rat VGLUT2, forming synaptic contact with GFP cells, suggesting the presence of iGABAPs graft synapses with host excitatory neurons (Fig. 6d). This evidence of bidirectional host–graft synaptic integration was rarely observed in DSNPs grafts (Fig. 6b–d). In lower spinal segments, caudal regions away from the iGABAPs graft, we can detect a higher number of mCherry+ cells colocalizing with host inhibitory (GABA^+^) interneurons in the dorsal horns, reflecting long-range projections synapsing with local spinal circuits (Fig. 6e). Consistent with limited recovery of connectivity, mCherry+ signals in lumbar regions were minimal in the DSNPs group at this time point. These findings indicate that grafted iGABAPs receive inputs from upstream cortical control centers and distant local neurons, which further form synaptic transmission to the lower spinal segments, indicating effective integration into both long-projecting and local spinal cord circuitries.

### iGABAPs grafts improve CNP and locomotion activity after contusive SCI

**Fig. 7:**
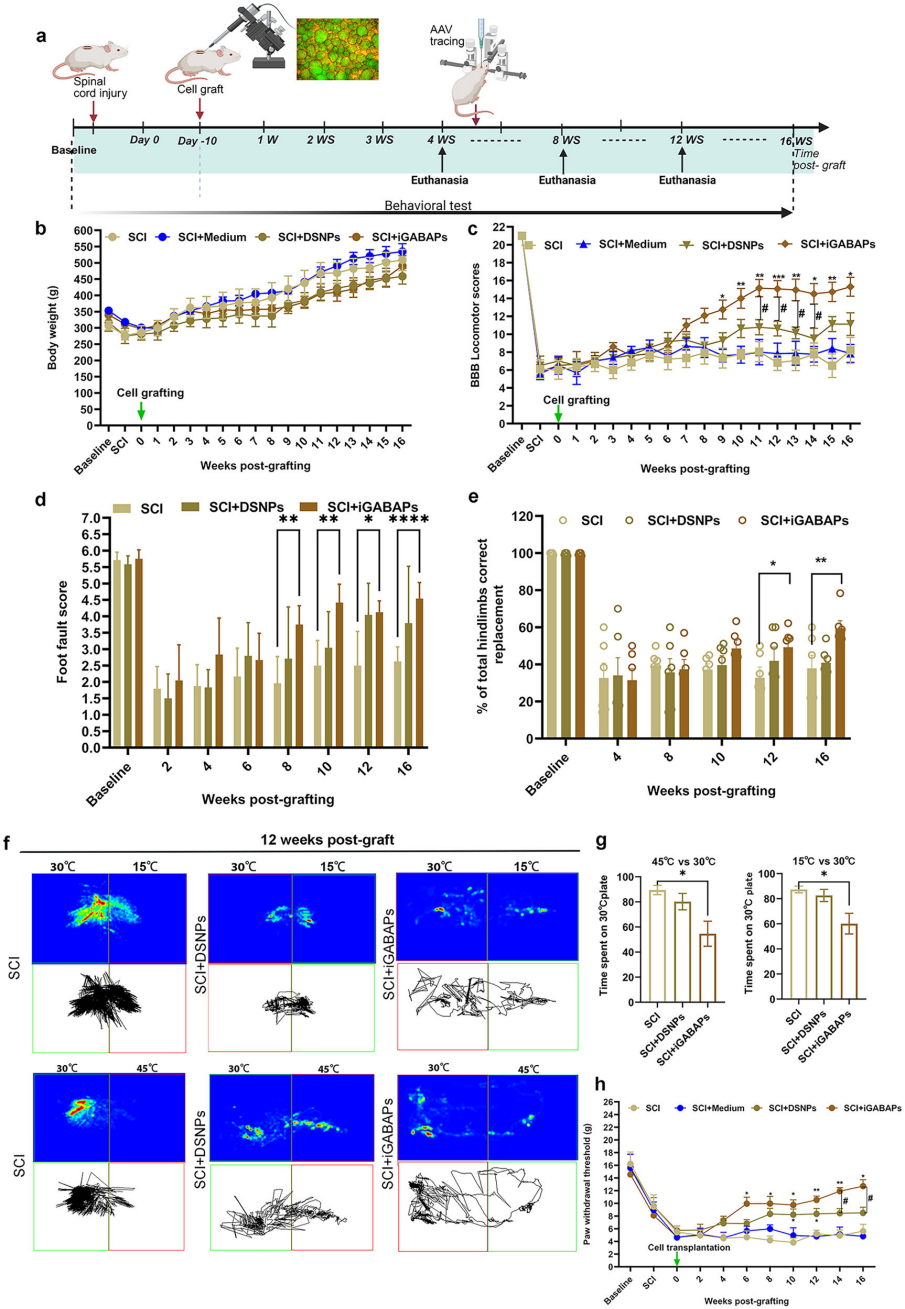
Significant functional improvement after transplantation of iGABAPs into contusive SCI. **a** Schematic diagram showing the time course for functional analysis after SCI with cell transplantation. **b** Time course analysis of body weight in SCI and SCI with vehicle injections, DSNPs grafts and iGABAPs grafts. **c** BBB scores of SCI and SCI with vehicle injections, DSNPs grafts and iGABAPs grafts. Two-way repeated-measures ANOVA with Bonferroni’s post hoc test. ^*^ or ^#^*P* < 0.05, ^**^P < 0.01. **d** Grid walk quantitative analysis is measured as a percentage of hind limb placement. One-way ANOVA with Tukey’s post hoc test; ^*^P < 0.05, ^**^P < 0.01, ^****^P < 0.0001. **e** Foot fault score analysis of hind limb measured by rating scale for foot placement in the skilled ladder rung walking test (correct placement 6 points, partial placement 5 points, placement correction 4 points, replacement 3 points, slight slip 2 points, deep slip 1 point and total miss 0 points). One-way ANOVA with Tukey’s post hoc test; ^*^P < 0.05, ^**^P < 0.01. **f** Representative track imaging of the two-plate preference test at 30 °C versus 15 °C or 30 °C versus 45 °C in SCI and SCI with DSNPs and iGABAPs grafts at 12 weeks after grafting. **g** Occupancy quantification of 30 °C versus 15 °C or 30 °C versus 45 °C in SCI and SCI with iGABAPs grafts. Two-tailed unpaired Student’s *t*-test, ^*^P < 0.05. **h** Time course of the mechanical allodynia, as measured by the von Frey force threshold for withdrawal in SCI and SCI with iGABAPs grafts. Two-way repeated-measures ANOVA with Bonferroni’s post hoc test. ^*^or ^#^*P* < 0.05, ^**^P < 0.01. ^*^ versus SCI; ^#^: SCI + iGABAPs versus SCI + DSNPs. All data are expressed as mean ± s.e.m.

To evaluate the therapeutic potential of iGABAPs grafts in SCI, we performed sensory and motor functional tests in graft animals, comparing them with nongrafted controls (Fig. 7a). Body weight remained stable across all groups, with no significant differences detected (Fig. 7b). We further evaluated the hind limb locomotion by the BBB locomotor scale method before injury and after injury weekly. Immediately after injury, BBB scores dropped to a mean of <7 in all SCI rats, and partial recovery was observed by 1 week after injury, with scores stabilizing at around 8. The iGABAPs recipients demonstrated a significant increase in BBB score from 9 weeks after grafting onward compared with the SCI and SCI + vehicle groups. A significant advantage over the DSNPs group became apparent at 11 weeks (Fig. 7c). The grid-walking test evaluates skilled locomotor function by counting the percentage of correct steps on paw replacement and foot faults as rats traverse the metal grid^22^. Compared with nongrafted controls, iGAPAB-grafted rats exhibited significantly fewer hind limb foot faults from 8 weeks after grafting and demonstrated improved paw placement accuracy by 12 weeks after grafting (Fig. 7d,e). No such improvement was observed in the DSNPs group. Remarkably, iGABAPs transplantation demonstrated significant and early relief from CNP. In the two-temperature preference test, grafted animals spent more time on the 45 °C (heat) and 15 °C (cold) surfaces, whereas SCI controls predominantly remained on the 30 °C plate, indicating reduced thermal hypersensitivity. No significant thermal sensitivity was observed in SCI with DSNPs graft compared with SCI (Fig. 7f,g). Furthermore, iGABAPs grafts markedly attenuated mechanical allodynia from 6 weeks after grafting, with benefits sustained through the 16-week endpoint (Fig. 7h). By contrast, the DSNPs group showed a later and more modest effect, with differences emerging only at 10 and 12 weeks. This early amelioration of CNP is probably mediated by rapid neuromodulation and environmental modification by GABA that is produced from iGABAPs grafts. Collectively, these findings demonstrate the superior therapeutic potential of iGABAPs grafts for restoring locomotor function and alleviating chronic pain after SCI.

## Discussion

In this study, we screened different TF combinations, *P* (*PTF1A*), *PA*, *PLA*, *PLAp* and *BPLA*, for rapid generation of GABAergic progenitors with dorsal spinal cord identity from human PS cells. With defined TF combinations, in particular the *PLA*, cells activated intrinsic differentiation programs, showing remarkable adaptation to the deleterious injury niche, which exhibited enhanced survival, engraftment and maturation, effectively forming new relay circuits with the host. iGABAPs grafts also dramatically improved injury conditions by reducing endogenous apoptosis and glial scarring, preserving host neurogenesis during the subacute SCI stage. The combined beneficial effects of iGABAPs grafts effectively improved somatosensory and locomotor functions in a traumatic injury model.

The significant loss of GABAergic interneuron function in the dorsal horn following traumatic SCI disrupts GABA release. This deficiency of inhibitory neurotransmission, in particular GABA, often results in glutamatergic neuronal hyperexcitability and excitotoxic levels of glutamate, leading to CNP and secondary injury, including progressive neuronal and glial cell death^3–5,7^. A meta-analysis of SCI cases revealed an overall prevalence estimate of 53% for neuropathic pain development, which in most cases is CNP^2^. CNP has a profound and negative impact on quality of life without effective treatments. Notably, the sensitivity of the nociceptive system may change as a result of sustained CNP, leading to refractoriness to interventions^2,6^. In addition, GABA was also shown to exert a neuroprotective nature in reducing excitotoxicity and promoting neurite outgrowth in response to central nervous system damage^8^; thus, restoring functional GABAergic neuronal properties in the dorsal spinal cord is critical to addressing SCI deterioration and its associated complications, particularly CNP. Nevertheless, current transplantation therapy of human neural progenitors derived from human PS cells does not favor differentiation and regeneration of GABAergic properties. The injury microenvironment was shown to be pro-astrocytic, which hinders neurogenesis and the maturation of human neural progenitor grafts into functional neurons^11,12,24,43^. Furthermore, graft hNSCs with spinal identity predominantly adopt excitatory neuronal fates, with a low percentage becoming GABAergic neurons^13,23^. Our study demonstrated that grafting iGABAPs induced by TFs in the injured spinal cord effectively differentiated into mature GABAergic neurons, showing significant mitigation of CNP symptoms as early as 4 weeks after grafting.

The superiority of a stepwise maturation of pure human GABAergic interneurons with brain characterizations by transiently overexpressing defined TFs such as ASCL1, DLX2 and LHX6 has been previously reported by several groups, which induce not only specification of brain GABAergic properties but also an accelerated maturation program^14–16^. It is worth noting that brain and dorsal spinal GABAergic neurons are regulated by different transcriptional signaling mechanisms. Although early studies demonstrated that transplanting mouse forebrain GABAergic neuron precursors from the medial ganglionic eminence into the adult spinal cord could reverse the persistent pain produced by peripheral nerve injury^10^, unmatched grafts with the recipient’s spinal tissue could potentially increase the complexity of the microenvironment^19^, limiting clinical applicability and therapeutic efficacy. In the spinal cord, the expression of *Ptf1a*, a bHLH TF, is restricted to dorsal GABAergic neural progenitor (dP4) and plays a central role in specifying GABAergic inhibitory neurons. *Ptf1a* activates downstream targets such as *Tfap2a/b* and *Prdm13*, which coordinate with *Lbx1* to induce *Pax2* and *Lhx1/5* expression for GABAergic neuron differentiation and maturation^30–34^. Conversely, *Ascl1* is required and sufficient for generating glutamatergic neurons from its dorsal progenitor dP5 in animal models^36^. Interestingly, *Ascl1* directly converts human PS cells or fibroblasts into glutamatergic neurons but can also generate brain GABAergic neurons when combined with different TFs, highlighting its functional discrepancy between humans and other vertebrates^14,37^. Our subsequent validation and screening in a human context identified several potential TFs, including *BRN2*, *PTF1A*, *ASCL1*, *PAX2* and *LBX1*, as key factors in determining and specifying dorsal spinal GABAergic identity. We confirmed that combinations of *PTF1A*, *LBX1* and *ASCL1* are sufficient to induce GABAergic progenitor colonies within 1 week of dox withdrawal. Enriched GABAergic mature neurons can be detected within 2 weeks of dox withdrawal, exhibiting robust expression of a GABA-sensing fluorescent reporter (GFP), elevated GABA levels measured by ELISA and functional electrophysiological properties.

Critically, our in vivo functional studies demonstrated the superiority and significant translational value of iGABAPs in treating SCI and its complications, CNP. Unlike DSNPs, which rely on extrinsic small molecules and growth factors for differentiation/maturation—a process often hampered by the inhibitory adult injury niche—iGABAPs are driven by intrinsic differentiation programs via overexpression of a specific set of TFs. This intrinsic program renders them less dependent on extrinsic neurotrophic support and more resilient to the deleterious injury environment. Consequently, iGABAPs grafts exhibited robust adaptation and superior integration into host neural circuits, demonstrated by rapid generation of mature GABAergic neurons with extensive long-range axonal outgrowth and forming functional synaptic connections with host. This enhanced integration may also be facilitated by the grafts’ matched anatomical identity with the host dorsal spinal cord, a property previously shown to promote the rewiring of lesioned circuits^13,20,23,44^. Notably, this accelerated maturation of GABAergic neurons is critically important for mitigating key secondary injury pathologies of SCI, particularly excitotoxic levels of glutamate, which induce neuronal hyperexcitability and progressive neuronal damage and apoptosis^1,3–5,7^. The GABA release could directly counteract this excitotoxicity by modulating glutamatergic hyperactivity and exerting broader neuroprotective effects^5,9^. This is supported by our findings that rapid generation of GABA-releasing neurons from iGABAPs grafts significantly reduced progressive apoptosis in host tissues and increased the survival of endogenous neuronal populations, and decreased CSPG expression, permitting axonal growth around and beyond the lesion site. Early amelioration of neuropathic pain observed by 6 weeks after grafting is probably mediated by rapid neuromodulation and environmental modification by GABA. Thus, the therapeutic superiority of iGABAPs stems from a synergistic mechanism: their cell-intrinsic program enables rapid GABAergic neuron generation and axonal growth, while their presence induces a permissive host environment that counteracts excitotoxicity and supports neuroprotection and circuit integration. This combined effect promotes significant functional recovery observed in both somatosensory and locomotor systems.

In conclusion, our findings provide a method for generating human dorsal spinal cord-derived GABAergic neurons for treating CNP in patients with SCI. This technology could facilitate faster maturation of specific cell types, reduce heterogeneity and mitigate the progressive deterioration of the spinal cord following injury. Previous research has shown that overexpressing five TFs—Foxg1, Sox2, Ascl1, Dlx5 and Lhx6—can rapidly convert mouse fibroblasts into functional forebrain GABAergic neurons^28^. Future studies should investigate whether similar or additional TF combinations can directly convert somatic cells into GABAergic spinal neurons, thereby enhancing therapeutic applications.

## Supplementary information

Supplementary Information

Supplementary Tables 1 and 2.

## Data Availability

All data generated or analysed during this study are included in this Article and its Supplementary Information.
