# Supplementary material for: Efficient generation of human dorsal spinal GABAergic progenitors for the treatment of spinal cord injury
Source: Exp Mol Med. 2026 Mar 6;58(3):832–47. doi: 10.1038/s12276-026-01665-8 (PMC13049108; doi:10.1038/s12276-026-01665-8)
Supplement: Supplementary file 1 — Supplementary Information [file 12276_2026_1665_MOESM1_ESM.pdf]

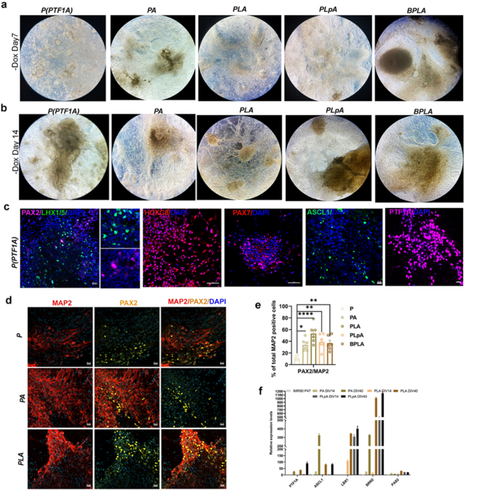

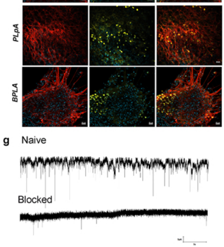

Supplementary Fig. 1. Right field imaging showing colony formation following overexpression of different TF combinations(PA, PLA, PLpA, and BPLA) at 1 week **(a)** and **(b)** 2 weeks after dox withdrawal. **(c)**Immunofluorescence staining for dorsal spinal progenitor markers, PAX7, HOXC8, PTF1A and ASCL1, in PTF1A-overexpressing hPSCs. DAPI was used to stain DNA in nuclei. **(d)** Immunofluorescence analysis of GABAergic neuronal markers(PAX2, MAPs) in hPSCs overexpressing TF combinations(PA, PLA, PLpA, and BPLA) at day 14 post-induction(-dox). Nuclei were stained with DAPI. **(e)**Quantification of the percentage of indicated markers as shown in (d). Data represent mean percentages ± SEM from three independent experiments. **(f)**Time course qPCR analysis of spinal dorsal identity and GABAergic differentiation markers in hPSCs overexpressing different TF combinations. Three independent experiments. **(g)**Spontaneous inhibitory postsynaptic potentials (IPSCs) recording of PLA induced GABAergic neurons, which were inhibited by adding Picrotoxin (PTX), a channel blocker of GABAA receptors.

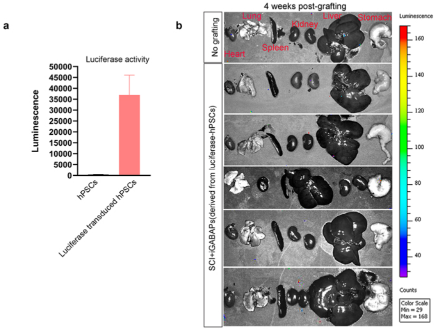

Supplementary Fig. 2. (a) Luminesence measurement for hPSCs and hPSCs labelled with lenti- CMV- Puro LUC, which contains the firefly luciferase gene. **(b)** Images of the bioluminescent signals record from different tissues isolated from non-grafting group and iGABAPs derived from Luciferase labelled hPSCs after 4 weeks post grafting

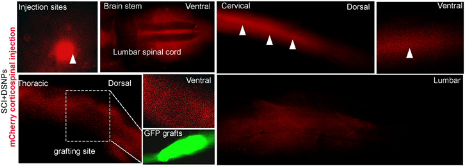

Supplementary Fig. 3. Representative images showing mCherry siganls in somatosensory and motor cortex(injection site) and trans-synaptic connection from cortical region to the brain stem (ventral and dorsal view), cervical and thoracic region of spinal cord, grafts at the lesion sites and caudal regions of the lesion, and Lumbar in DSNPs graft group. Arrows indicate strong positive mCherry signals.
