# Supplementary material for: Efficient generation of human dorsal spinal GABAergic progenitors for the treatment of spinal cord injury
Source: Exp Mol Med. 2026 Mar 6;58(3):832–47. doi: 10.1038/s12276-026-01665-8 (PMC13049108; doi:10.1038/s12276-026-01665-8)
Supplement: Supplementary file 2 — Supplementary Tables 1 and 2. [file 12276_2026_1665_MOESM2_ESM.docx]

**Table S1 List of primary and secondary antibodies**

| Primary antibody | Species | Dilution | Catalog No | Band |
| --- | --- | --- | --- | --- |
| STEM121 | Mouse | 1:200 | Y40410 | TaKaRa |
| TUJ1 | Mouse | 1:500 | ab2493 | Sigma |
| NeuN | Rabbit | 1:400 | Ab177487 | Abcam |
| NeuN | Mouse | 1:500 | MAB377 | Millipore |
| GFAP | Goat | 1:500 | Ab302644 | Sigma |
| PTF1A | Mouse | 1:200 | SC393011 | Santa Cruz |
| ASCL1 | Mouse | 1:200 | SC374104 | Santa Cruz |
| PAX7 | Mouse | 1:50 | AB_528428 | Developmental Studies Hybridoma Bank |
| HOXC8 | Mouse | 1:50 | AB_2618723 | Developmental Studies Hybridoma Bank |
| MAP2 | Mouse | 1:200 | M4403 | Sigma |
| MAP2 | Rabbit | 1:200 | 4542 | CST |
| GAD65 | Mouse | 1:200 | ab26113 | Abcam |
| LHX1/5 | Mouse | 1:50 | 4F2 | Developmental Studies Hybridoma Bank |
| GABA_ A | Rabbit | 1:200 | ab33299 | Abcam |
| GABA | Rabbit | 1:200 | A2052 | Millipore |
| PAX2 | Rabbit | 1:300 | 901001 | Biolegend |
| Anti-Vesicular Glutamate Transporter 1 Antibody | Guinea pig | 1:200 | AB5905 | Millipore |
| Anti-Neurofilament 70 kDa Antibody, clone DA2 | Mouse | 1:200 | MAB1615 | Millipore |
| Synaptophysin Antibody (EP10) | Mouse | 1:200 | NBP119222 | NovusBiologicals |
| IBA-1 | Rabbit | 1:200 | 016-26721 | Wako |
| CaMKII | Rabbit | 1:200 | ab52476 | Abcam |
| 5-HT | Goat | 1:200 | 20079 | ImmunoStar |
| Ki67 | Rabbit | 1:200 | GTX16667/SP6 | GeneTex |
| Ki67 | Mouse | 1:200 | M7240 | Dako |
| Caspase3 | Rabbit | 1:200 | 9662 | CST |
| CSPG | Rabbit | 1:200 | AB5320 | Millipore |

| Secondary antibody | Catalog No | Dilution | Band |
| --- | --- | --- | --- |
| Donkey anti rabbit 488 | A-11008 | 1:500 | Invitrogen |
| Donkey anti rabbit 568 | A10042 | 1:500 | Invitrogen |
| Donkey anti rabbit 647 | A-21247 | 1:500 | Invitrogen |
| Donkey anti guinea pig 568 | A-11075 | 1:500 | Invitrogen |
| Donkey anti mouse 647 | A-31571 | 1:500 | Invitrogen |
| Donkey anti mouse 488 | A-11001 | 1:500 | Invitrogen |
| Donkey anti mouse 568 | A-11011 | 1:500 | Invitrogen |

Table S2. 1 List of Primers

| GAPDH | ACATCAAGAAGGTGGTGAAGCAGG | AGCTTGACAAAGTGGTCGTTGAGG |
| --- | --- | --- |
| PTF1A | GAAGGTCATCATCTGCCATCGG | CCTTGAGTTGTTTTTCATCAGTCC |
| LBX1 | GCCTGCCTCTCCGCTCAC | ATCTCTTCGTCTTCCTCGTCCTC |
| ASCL1 | TCTCATCCTACTCGTCGGACGA | CTGCTTCCAAAGTCCATTCGCAC |
| PAX2 | ATCTGCATCCACCAACCCTG | GCTGAATCTCCAAGCCTCGT |
| SOX2 | GCTGCAAAAGAGAACACCAATCCC | AAACTTCCTGCAAAGCTCCTACCG |
| OCT4 | ATGCATTCAAACTGAGGTGCCTGC | CCCTTTGTGTTCCCAATTCCTTCC |
| NANOG | AGTATGGTTGGAGCCTAATCAGCG | ATCCTGGCTAACACAGTGAAACCC |
| PAX6 | GTACTGAATGACTCAACTGCTCGG | CTTTAGAAGGAAGCGACACTCTGC |
| PAX7 | GGAGGATGAAGCGGACAAGAAG | AGGTCAGGTTCCGACTCCACAT |
| NESTIN | CGTACAGGACCCTCCTGGA | TGAAAGCTGAGGGAAGTCTTGGAG |
| TUJ1 | AAGCCAGCAGTGTCTAAACCC | GGGAGGACGAGGCCATAAATA |
| MAP2 | AGGGCACCTATTCAAATACCAAAGAGAA | ACCTCCTCTGCTGTTTCTCTGTC |
| GAD1 | TGTCCAGGAAGCACCGCCATAA | TCCTTGACGAGAATGGCAGAGC |
| GAD2 | GCCAACTCTGTGACGTGGAATC | GCTGAAAGAGGTAGGAGGCATG |
| TLX3 | AGCCTCAACGACTCCATCCAG | GTGACAGCGGGAACCTTGG |
| OLIG2 | GTTCTCCCCTGAGGCTTTTC | GATAGTCGTCGCAGCTTTC |
| HOXB1 | GGTCAAGATTTGGTTCCAGAACCG | ATTGGTGGCTAGGTTCAGTTCAGG |
| EN1 | GGCGTCAACAACCTCACTGG | TGAACCTGTCCTTTGTGTATCTGC |
| FOXG1 | TCTAACAAGGTGTGGAGTGTCAGC | TACTGCACACATGGAAATCTGGCG |
| 36B4 | GTGATGTGCAGCTGATCAAGACT | GATGATCAGCCCGAAGGAGA |
| HOXB4 | AAAAAGAGAGACTCAGAGACCCGG | CTGGGAGGGGCACATTTTATTTCC |
| HOXC4 | GGGTGAATTTCAGGGGAAATGAGG | CTCAAACTGAACAGCTCTGAGAGG |
| HOXC6 | TTAGCACCGTCAGTGTTCCTATCC | TATACAGGAGGGTAACACGAAGGG |
| HOXC8 | AGGAACCTGATGGAAACCTGAAGG | ATCAAACAGCGAAGGAGAGGAAGG |
| HOXC9 | TAGAGTTAGTTCTACCCAGCGAGG | ACCTGGACCAAATACGATACAGGG |
| HOXC10 | CTCACACACAGCATTCTGTTCTCC | ACACGAACACTAGCCGAACTTTCC |
| HOXB8 | CGTGGATCTCCTTCCCTTCT | GAATTACGGCGTGAATAGGC |
| HOXD8 | CCGCGAAGTTTTACGGATAC | GGAGCTGCTTGTGGTCTCAT |
